# Supplementary material for: Creep fronts and complexity in laboratory earthquake sequences illuminate delayed earthquake triggering
Source: Nat Commun. 2022 Nov 11;13:6839. doi: 10.1038/s41467-022-34397-0 (PMC9652330; doi:10.1038/s41467-022-34397-0)
Supplement: Supplementary file 1 — Supplementary Information [file 41467_2022_34397_MOESM1_ESM.pdf]

Supplementary Materials for

Creep Fronts and Complexity in Laboratory Earthquake Sequences Illuminate Delayed Earthquake Triggering

Sara Beth L. Cebry, Chun-Yu Ke, Srisharan Shreedharan, Chris Marone, David S. Kammer, Gregory C. McLaskey

Correspondence to: gcm8@cornell.edu

This file includes: Supplementary Figs. 1 to 18, Supplementary Tables 1 to 2.

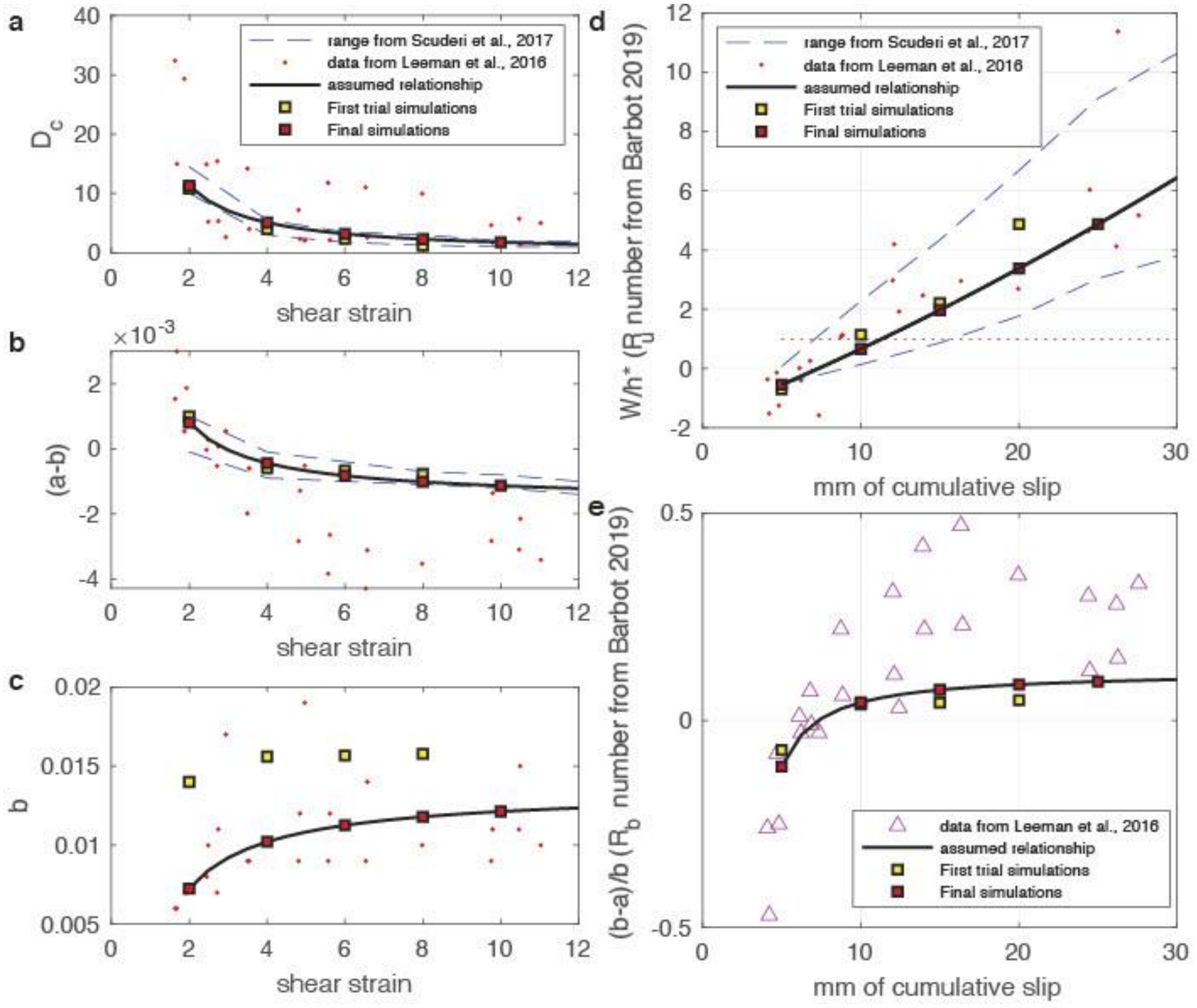

Supplementary Fig. 1. Evolution of friction parameters with shear strain. The friction parameters assumed in our numerical models (red squares) are designed to generally match laboratory data. Values are listed in Extended Data Table 1. Cumulative strain is related to slip by assuming 2.5 mm layer thickness. Simulations with parameters that differ only in the value of b (yellow squares) showed similar behavior, with a somewhat greater propensity for creep fronts.

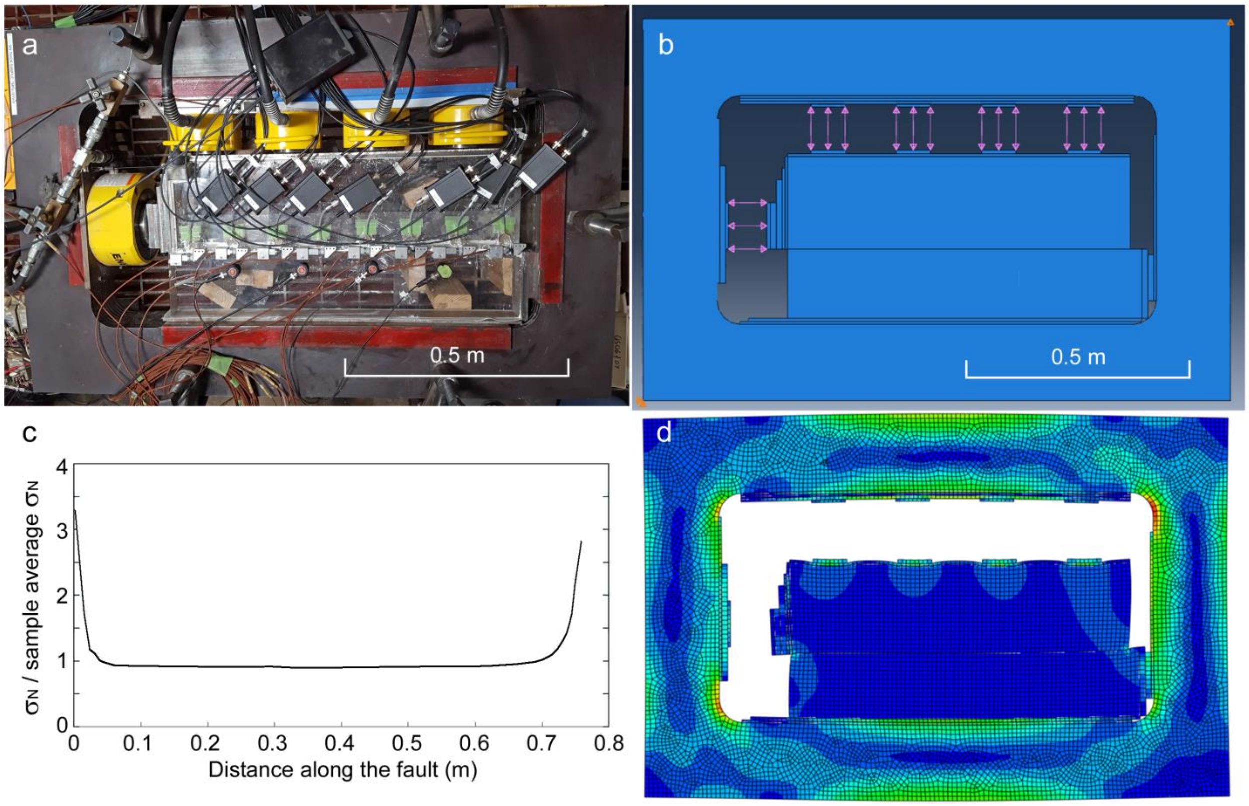

Supplementary Fig. 2 a, Photograph of the laboratory experiment with strain gage array installed. **b**, Corresponding finite element model of the PMMA sample in the steel frame to estimate the distribution of shear and normal stress on the laboratory fault. No interfaces are allowed to slip. Force is applied at the locations of the hydraulic cylinders C1-C5 to simulate a sample average normal stress of 7.75 MPa and sample average shear stress of 6.8 MPa. **c**, Results of the finite element model show the distribution of normal stress along the fault relative to the sample average normal stress. **d**, Mesh geometry, exaggerated deformation, and Mises stresses (colors) from the same model.

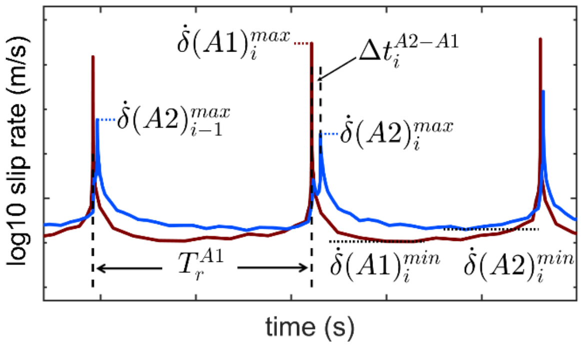

Supplementary Fig. 3. Definitions of parameters. The y axis is log of slip rate. The red and blue lines are the slip rated measured at A1 and A2, respectively. The A1 recurrence time is T_r^{A1}. There is typically one A2 event each A1 cycle at a time $\Delta t^{A2\text{-}A1}$ after the A1 event. The maximum and minimum slip rate at A1 and A2 is calculated each A1 cycle.

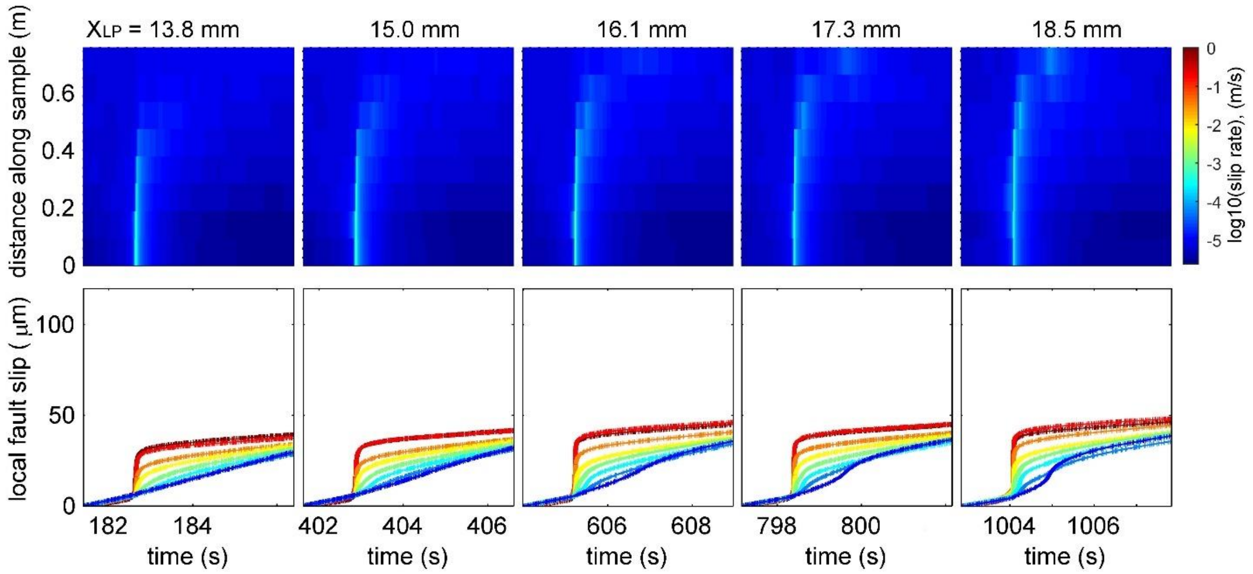

Supplementary Fig. 4. Examples of how creep fronts extending from A1 ruptures become more defined with increasing cumulative displacement x_{LP} from 13 mm to 19 mm. Upper panels show the slip rate as a function of space and time. Lower panels show, on the same time scale, the original slip data obtained from the eddy current displacement sensors used to generate the upper panels. Eddy current sensor locations are shown in Fig. 1a.

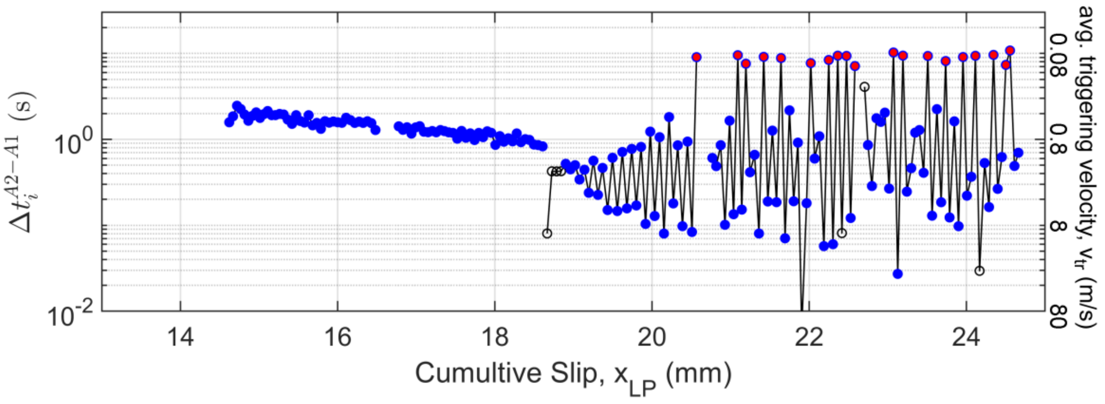

Supplementary Fig. 5. Triggering velocity throughout the experiment (see Fig 1). The time between A1 and A2 events $\Delta t^{A2\text{-}A1}$ decreases with shear displacement (x_{LP} =14.5 to 18.5 mm) indicating that creep fronts grow systematically faster and/or A2 event nucleate faster with increasing shear. At x_{LP} =19 mm, the behavior oscillates between progressively longer and shorter triggering times. Magenta circles indicate ($\Delta t^{A2\text{-}A1} > 0.9T_r^{A1}$) in which triggering is so slow that a second A1 event occurs before an A2 event occurs. Open circles indicate cycles where data are likely influenced by other factors (simultaneous nucleation of A1 and A2, or first few events after an unload-reload cycle).

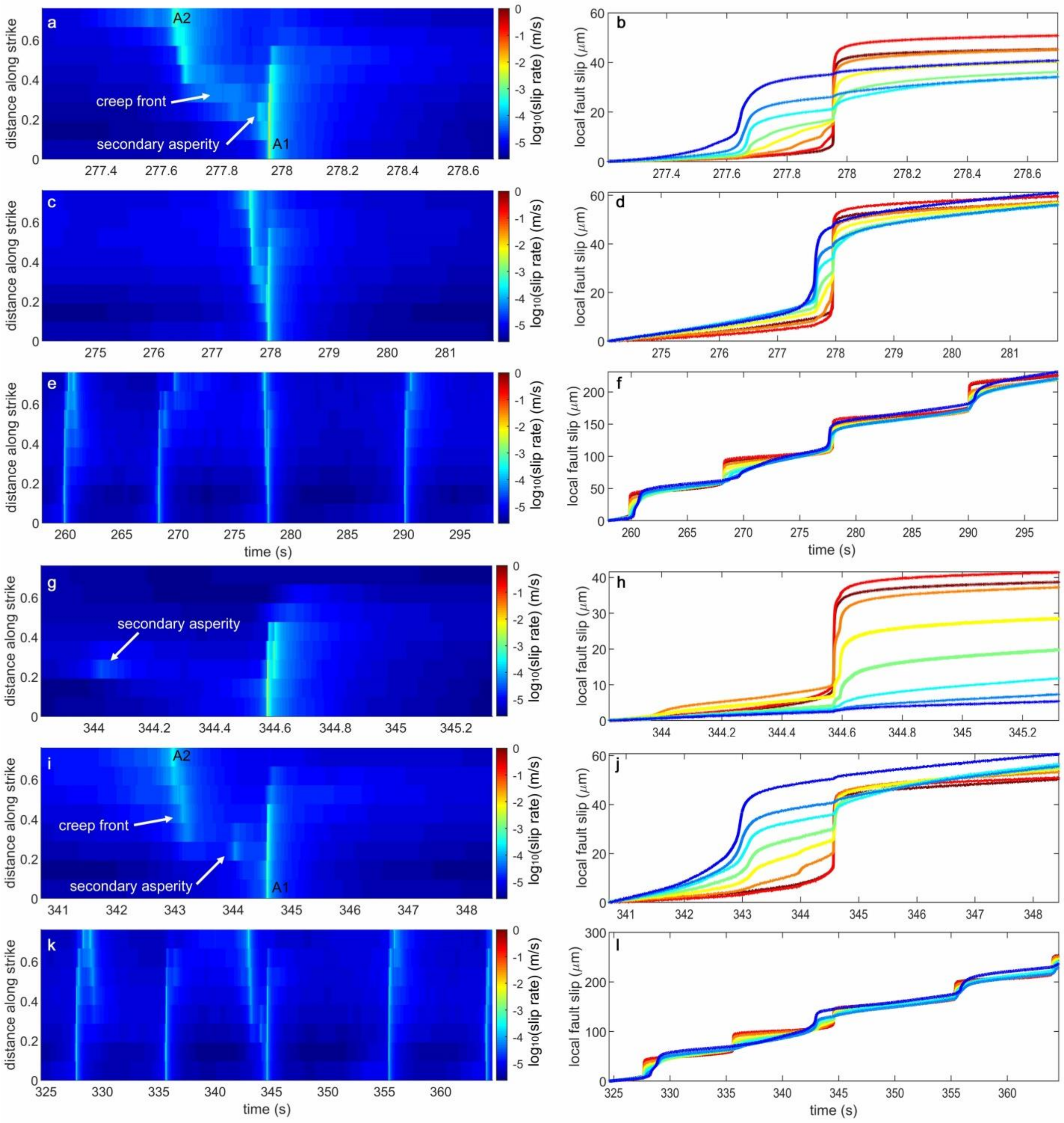

Supplementary Figure 6. Two examples of A2-to-A1 creep fronts. a-f, Left panels show the slip rate as a function of space and time at three different time scales. Right panels show the original slip data obtained from the eddy current displacement sensors used to generate the panels on the left. Eddy current sensor locations are shown in Fig. 1a. **g-l**, An example of a slower creep front that occurs later in the sequence. All plotting parameters are identical to **a-f**.

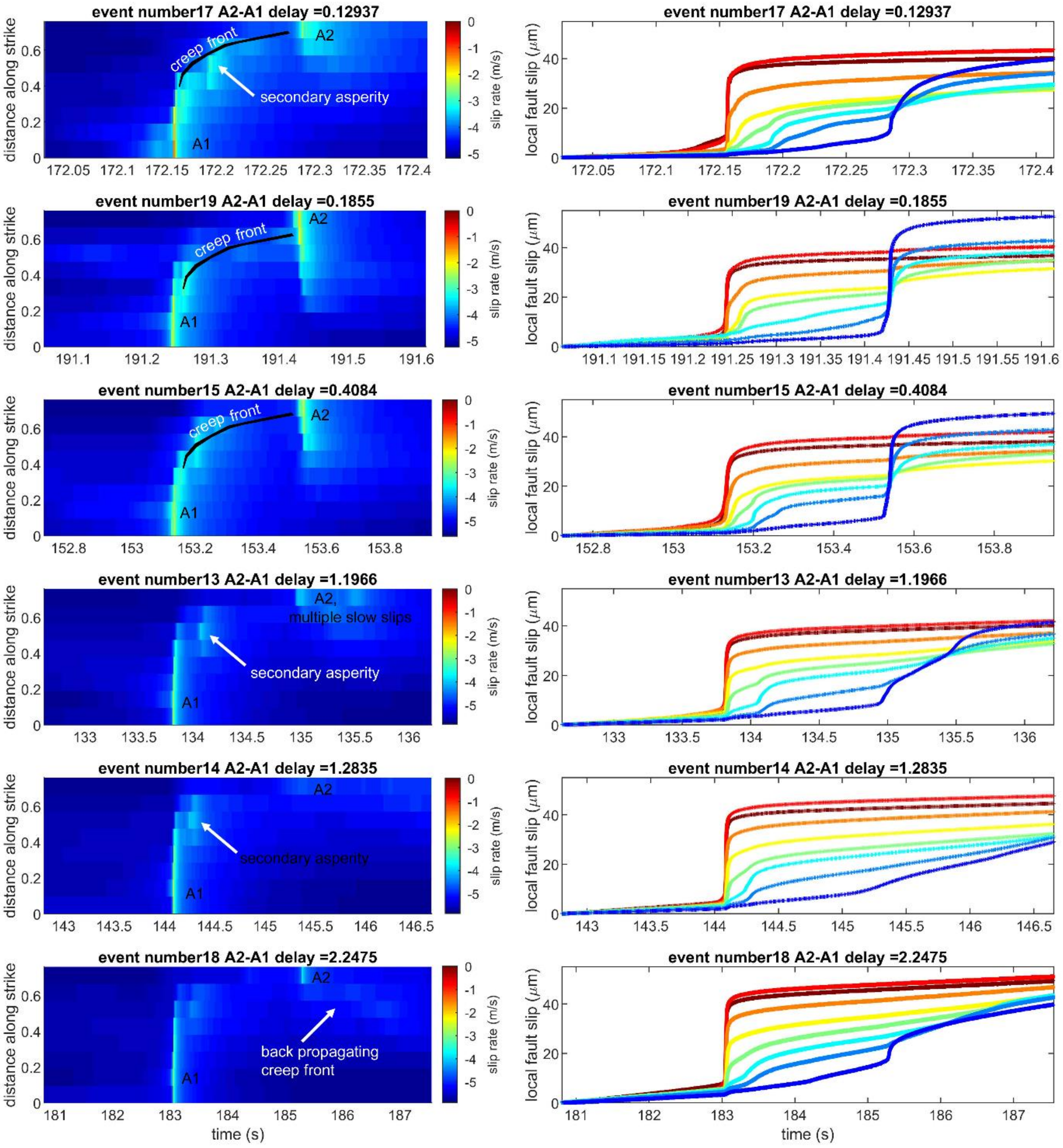

Supplementary Fig. 7. Examples of the variable behavior of the sample at x_{LP} = 23 mm. Events are ordered based on $v_{tr} = W/\Delta t^{A2\text{-}A1}$ (fastest on top, slowest on bottom), and the time window is scaled by $\Delta t^{A2\text{-}A1}$. This comparison shows that, in general, faster creep fronts are correlated with faster, stronger A2 events. A1, A2, creep fronts, and secondary asperities are annotated on some panels. Eddy current sensor locations are shown in Figure 1a.

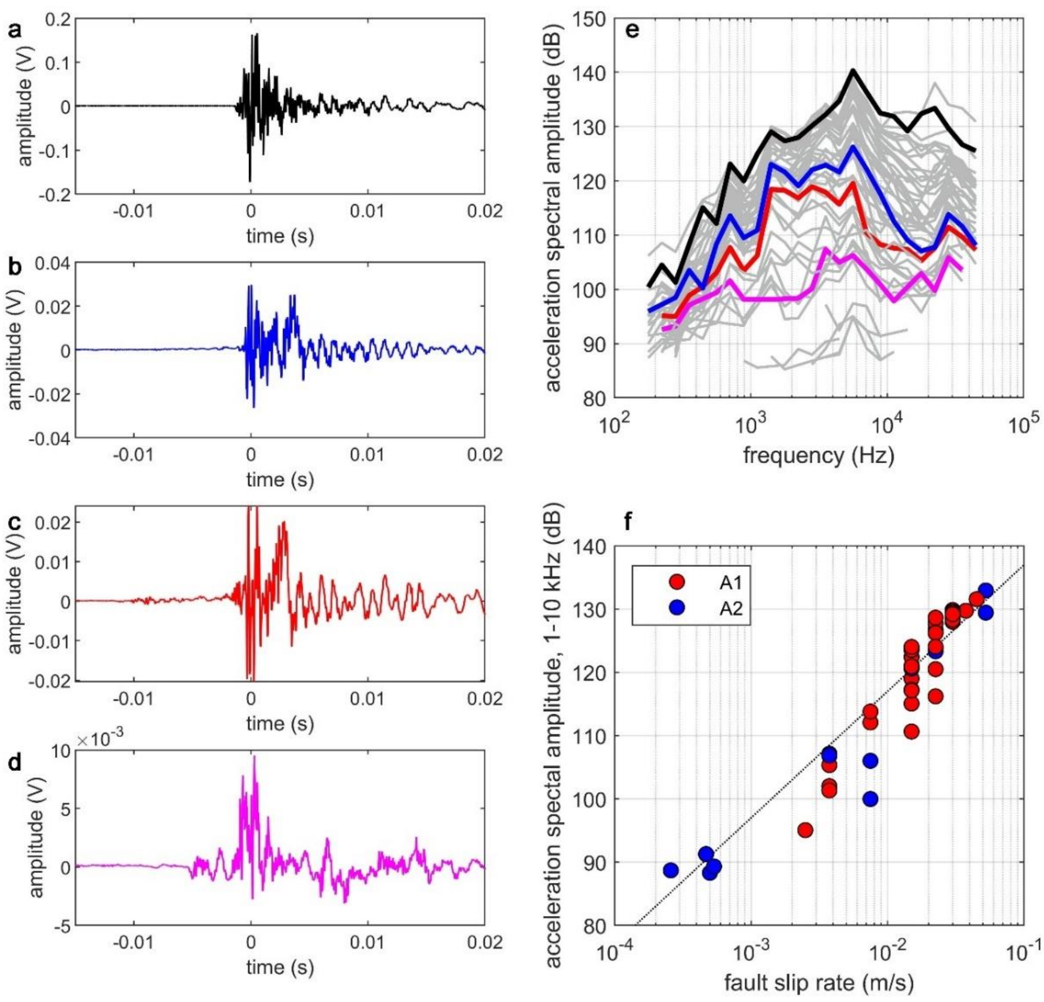

Supplementary Fig. 8. Measured ground motions, spectra, and slip rate for the laboratory earthquakes. a-d, example vertical ground motions measured on the top surface of the stationary block, 380mm off the fault (Figure 1) from four different A1 events ranging from large (a) to small (d) (note differences in amplitude scale). **e**, acceleration spectral amplitudes from the four events of a-d (colors) against spectra from other events from 22.5 mm < x_{LP} < 24 mm. **f**, average acceleration spectral amplitude is correlated to the maximum slip rate $\dot{\delta}_{\max}$ measured from the slip sensor closest to the asperity that ruptured (A1 or A2).

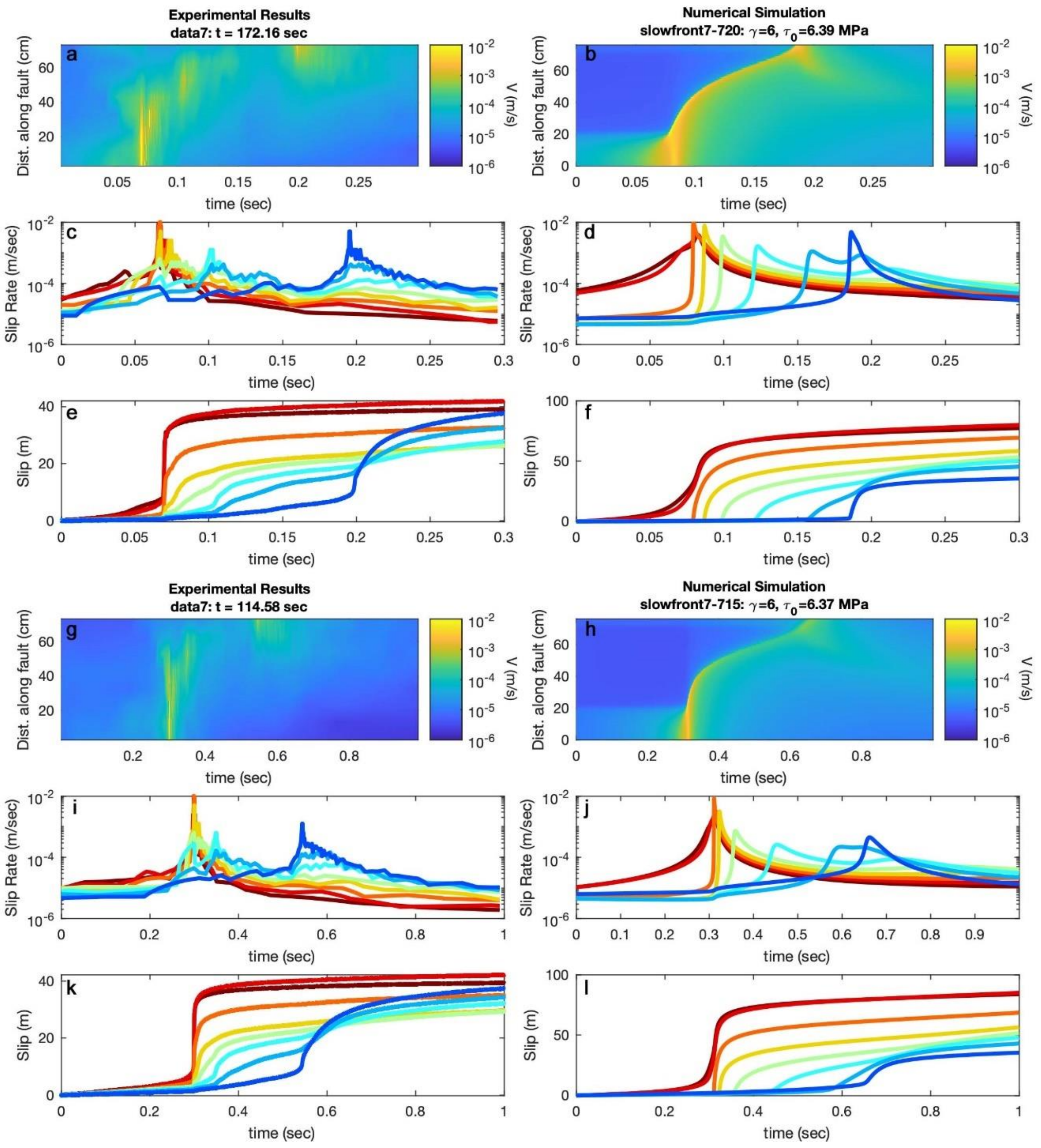

Supplementary Fig. 9 Direct comparisons between experimental observation (**a**, **c**, **e**, **g**, **i**, **k**) and numerical simulation (**b, d, f, h, j, l**). Example 1 (**a-f)** is somewhat faster than example 2 (**g-l**). Slip rate as a function of space and time is shown in **a-b**, **g-h**, slip rate at 8 locations is shown in **c-d**, **i-j**, and slip at those same 8 locations is shown in **e-f**, **k-l**. Slip sensor locations are shown in Fig. 1a.

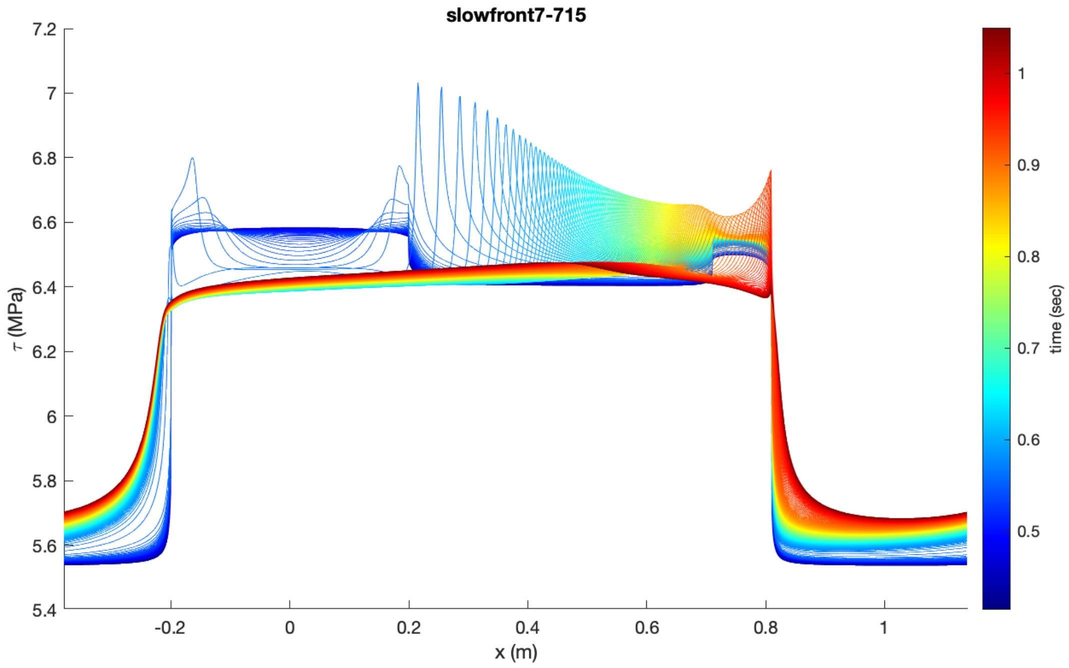

Supplementary Fig. 10. Snapshots of shear stress as a function of position for the simulation shown in Supplementary Fig. 9 (g-l). Note that the creep front is a strong stress concentration propagating outward from A1 (x = 0 m) from left to right. The stress concentration diminishes as the creep front slows.

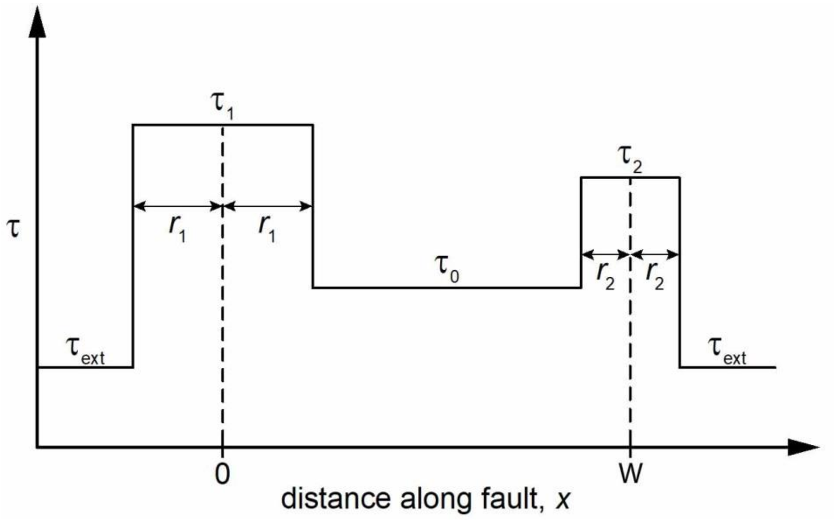

Supplementary Fig. 11: Initial shear stress distribution in simulations. The domain of interest is defined as 0 <= *x* <= W, where W is the fault length, 0.76 m. τ_1, τ_2, and τ_0 are the shear stress at the Asperity 1, Asperity 2, and in between, respectively. r_1 and r_2 are the radius of Asperity 1 and Asperity 2, which are 0.2 m and 0.05 m, respectively. τ_{ext} is the initial stress outside the domain of interest. It is set to be lower than the shear stress in the domain of interest so that the external region keeps creeping uniformly close to the initial velocity during the simulation.

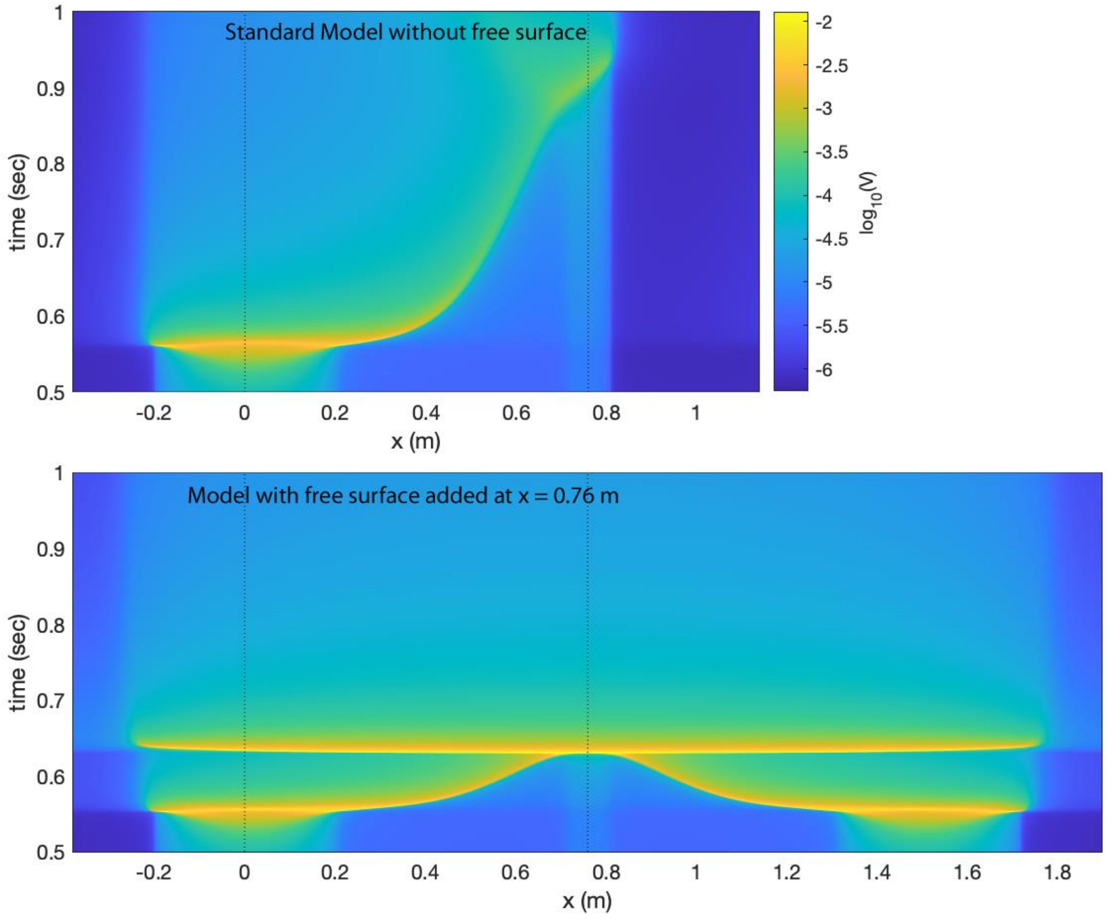

Supplementary Fig. 12 Effects of free surface in numerical models show that the free surface causes an increase in speed of the creep front and a faster, stronger rupture of the asperity which causes a strong back-propagating rupture. These differences between the model and experiment explain why far larger asperity sizes (r_1, r_2) were needed in the model, compared to asperities that were highly localized near the sample ends in the experiment (see Supplementary Fig. 2). These differences affect the precise speed of the creep fronts, and relative strength and size of the A1 and A2 asperities required to produce the delayed triggering but not the qualitative behavior that we report in this work: that creep fronts act to produce the delayed triggering observed, and that creep front propagation speed is highly sensitive to initial stress levels.

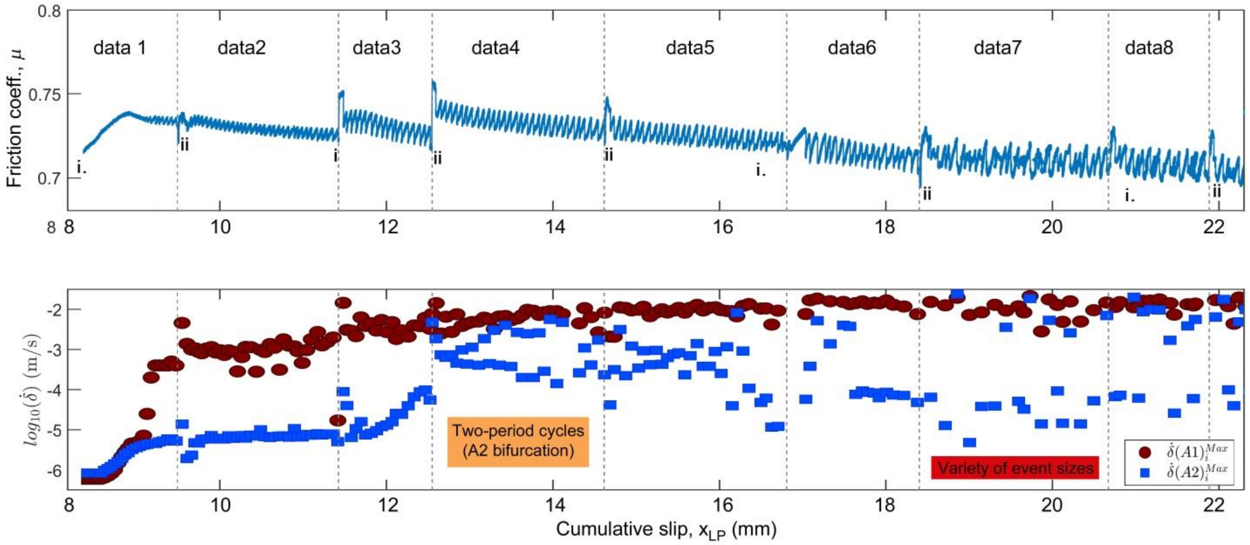

Supplementary Figure 13. Evolution of behavior in a second experiment. The upper panel shows the evolution of friction coefficient with cumulative fault slip. Similar to Fig. 1c, the annotations mark unload-reload cycles (i) or holds (ii) where the sample rested in essentially stationary contact. The lower panel shows the maximum slip velocity at A1 (red) and A2 (blue) every A1 slip cycle, similar to Figure 1d. This second experiment reproduces all the main observables as the first experiment including steady sliding and strengthening for x_{LP} < 8.5 mm, development of slow slip events on A1 at x_{LP} = 9 mm, the A2 bifurcation where A2 events oscillated between slow and progressively faster events at x_{LP} = 13 mm, and complex interactions between A1 and A2 at x_{LP} > 14 mm. However, in this second experiment A2 generally produced faster slip events with larger stress drop. While A2-to-A1 creep fronts (opposite their usual direction) occurred only occasionally in the first experiment, they occurred frequently in the second. Behavioral transitions (from steady sliding to slow slip to faster slip) also occurred at somewhat smaller x_{LP}, likely due to a somewhat thinner initial gouge layer thickness and/or stronger asperities. Stronger heterogeneity causes the behavioral progression to occur at smaller R_u levels (smaller X_{LP}) compared to a homogenous fault, so the stronger A2 events of this experiment likely decreased the X_{LP} at which behavioral transitions occurred compared to the first experiment.

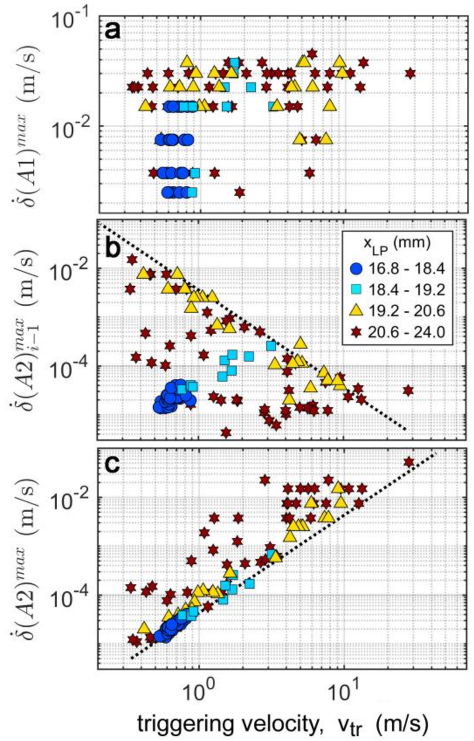

Supplementary Fig. 14. Triggering velocity changes with strength of previous events.
a, The A1-to-A2 triggering velocities v_{tr} are not well correlated with the strength of the A1 events that initiated them. **b**, Instead, v_{tr} is correlated with the strength of the previous A2 event. **c**, v_{tr} is also correlated with the strength of A2 events it triggers. The timing of the events shows that highly repeatable, characteristic events (blue circles) transition (cyan squares) to variable but predictable events (yellow triangles) that oscillate back and forth between slow and fast. With continued fault slip, interactions between creep fronts and the A1 stick-slip cycle produce more varied behavior (red stars) that are bounded by dashed lines which indicate relationships $\dot{\delta}_{\max} \sim v_{tr}^{-2}$ and $\dot{\delta}_{\max} \sim v_{tr}^{2}$ in b and c, respectively. We use maximum slip rate ($\dot{\delta}_{\max}$) as a proxy for the strength of the earthquakes since it is linearly related to static stress drop[44] and the measured acceleration spectral amplitude in the 1-10 kHz band (Supplementary Fig. 8).

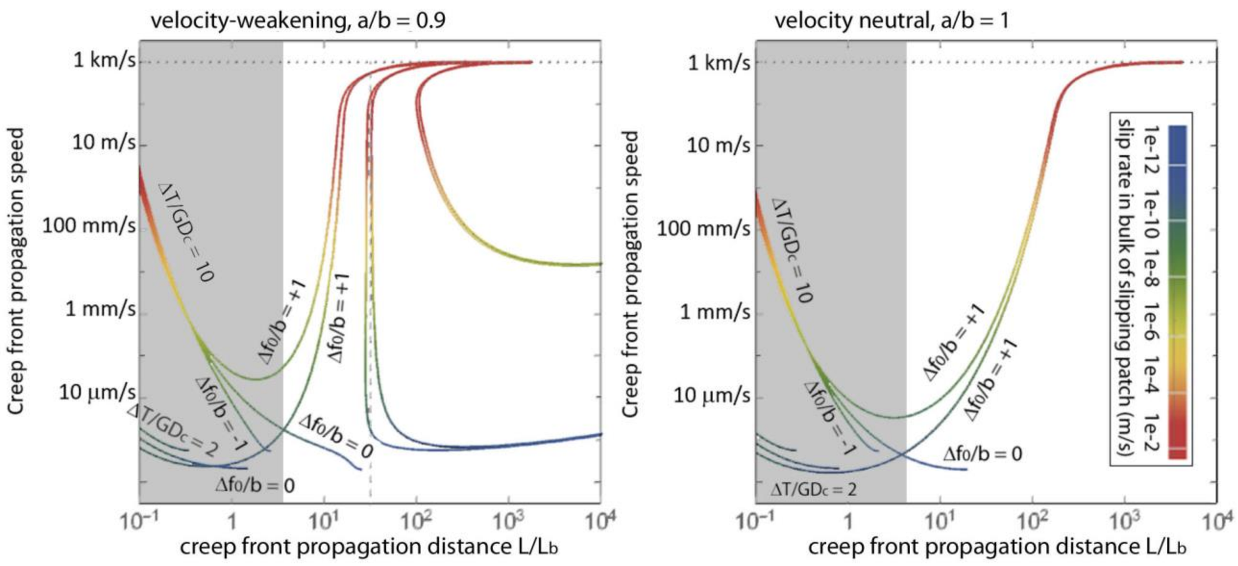

Supplementary Fig. 15 Adapted from Figure S5 of Garagash (2021) using RSF with aging law. Crossover from hypocentral forcing-dominated creep fronts (grey background) at small propagation distance L/L_b to initial overstress-dominated creep fronts (white background) at larger propagation distances. This behavior occurs both for velocity weakening rheology (left panel), velocity neutral rheology (right panel), and, to a lesser extent, with velocity strengthening rheology (not shown). Initial fault overstress is quantified by $\Delta f_0/b$ and the hypocentral forcing is quantified by $\Delta T/GD_c$ where ΔT is the hypocentral Coulomb forcing. Garagash (2021) studied creep fronts resulting from either constant volume fluid injection (constant ΔT) or constant volumetric injection rate (constant rate of ΔT). The creep front dynamics resulting from discrete seismic ruptures, as studied here, are best represented by the constant ΔT case.

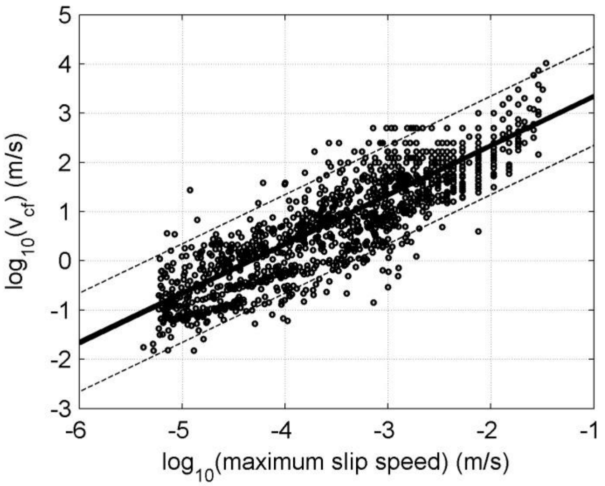

Supplementary Fig. 16. Creep front propagation velocity against slip velocity, similar to Figure 4 of Ariyoshi et al., (2019). Each circle corresponds to a measurement made from an adjacent pair of slip sensors for a propagating creep front. The propagation velocity v_{cf} is determined from the expression $v_{cf} = d/(t^{i+1} - t^{i})$ where d = 0.1 m is the sensor spacing and t^i is the time of the maximum slip velocity recorded at sensor i. The maximum slip speed associated with each measurement is taken as the average of the maximum slip speeds measured at the two adjacent sensors that were used to calculate v_{cf}. The bold line is the theoretical relation: $v_{cf} = G/\Delta\tau * v_{slip}^{max}$, where v_{slip}^{max} is the maximum slip speed upon propagation of the creep front, G = 1.1 GPa is the shear modulus and $\Delta\tau$ is the shear stress drop upon passage of the creep front, which is taken here as 50 kPa, consistent with average values shown in Fig. 4a.

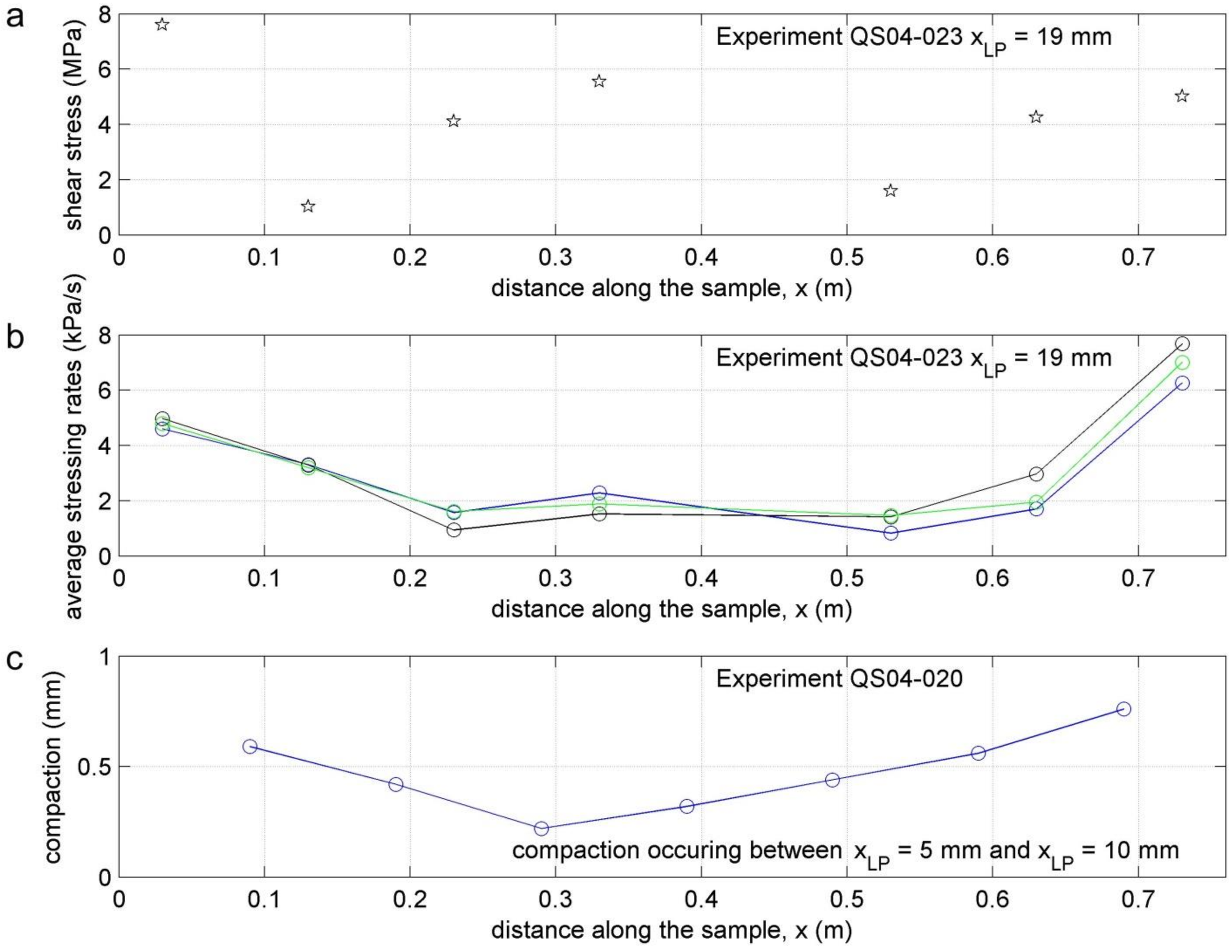

Supplementary Fig. 17. Distribution of shear stress, shear stressing rate, and compaction along the sample. (a) Absolute shear stress measurements were made using a reference strain measurement when sample is not loaded. These measurements may not be entirely reliable because the strain and reference strain measurements were made many hours apart. (b) Shear stressing rate estimated over ≈10 s time intervals when the sample was being loaded and no slip events occurred. Different colors correspond to different time intervals to show stability over many stick-slip cycles. The faster rates at the sample ends indicate that more shear stress accumulates there and that the sample ends are stronger (they are able to carry more shear stress than the center of the sample). (c) Compaction measurements made by comparing gouge layer thickness after 5 mm of cumulative slip to that after 10 mm of cumulative slip at 10 MPa sample average normal stress. This shows that the ends of the sample compacted more than the center of the sample, consistent with the higher normal stress there. Note that the compaction measurements were made in a different experiment (QS04-020) with similar conditions, described in Supplementary Table 2.

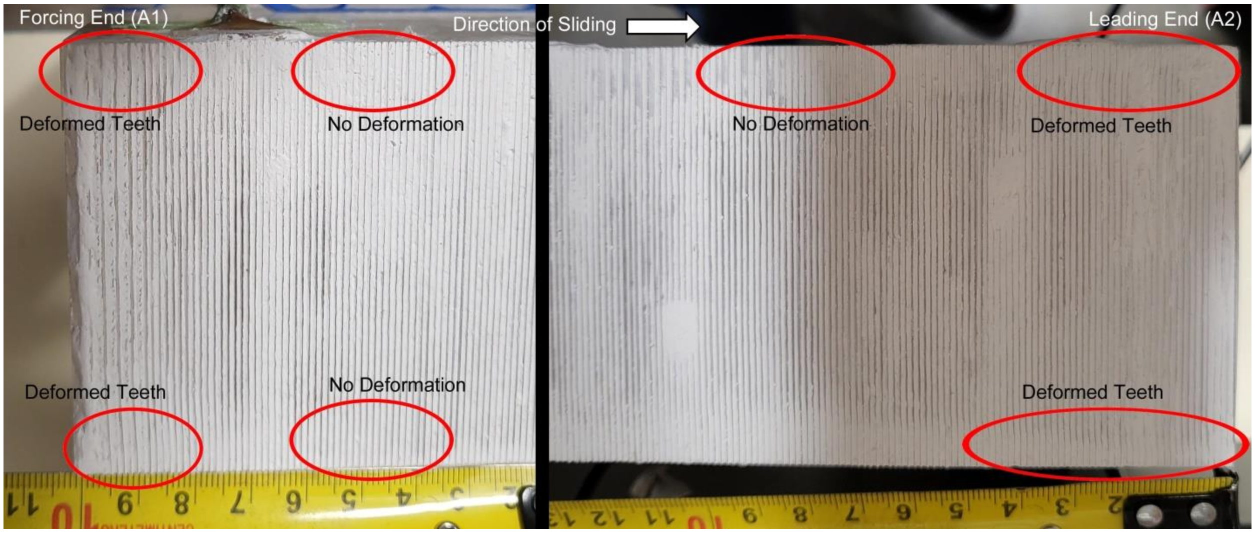

Supplementary Fig. 18. Annotated photographs of the forcing end (left) and leading end (right) of the moving block taken after the experiment was completed, the two sample halves were separated, and the majority of the gouge was brushed off the fault (photo taken after experiment QS04_023, see Supplementary Table 2). The teeth machined into the PMMA block can be seen as vertical lines. Compacted quartz gouge (white) is still stuck between the teeth, which is an indication that slip occurred within the gouge layer and not at the gouge/PMMA interface. There is evidence of plastic deformation of the PMMA teeth within 20-50 mm of the sample ends, while no deformation is observed closer to the center of the sample. The plastic deformation indicates that both shear and normal stress levels were higher near the sample ends (A1, and A2) than in the center of the sample. Note that the deformation reported here is the result of the cumulative effect of all experiments reported in Supplementary Table 2.

Supplementary Table 1

Slip independent Parameters	Units	Values				
sample length, W	m	0.76				
normal stress, σ_N	MPa	10				
shear modulus, G	GPa	1.1				
Poisson's ratio, ν	n/a	0.35				
$G' = G/(1-\nu)$	GPa	1.69				
**Slip dependent parameters**						
load point displacement, X_{LP}	mm	5	10	15	20	25
shear strain	n/a	2	4	6	8	10
b	n/a	0.00726	0.01021	0.01125	0.01179	0.01213
D_c	microns	11.266	5.0766	3.1847	2.2876	1.7699
b-a	n/a	-0.00081	0.00044	0.00082	0.00101	0.00114
a	n/a	0.00807	0.00977	0.01043	0.01078	0.01099
a/b	n/a	1.111	0.957	0.927	0.914	0.906
$h^* = 2D_cG'/(\pi\sigma_N (b-a))$	m	-1.507	1.239	0.416	0.243	0.167
$L_b = D_cG'/(\sigma_N b)$	m	0.263	0.084	0.048	0.033	0.025
W / h*	n/a	-0.50	0.61	1.83	3.13	4.54
$L/L_b = W/2/L_b$	n/a	1.45	4.52	7.93	11.57	15.39

Supplementary Table 2:

Experiment	Date	Gouge Type	Gouge Thickness (as prepared)	Normal Stress
QS04_001	7/14/2018	gypsum	3-5 mm	1, 7, 14 MPa
QS04_002	7/15/2018	gypsum	3-5 mm	7 MPa
QS04_003	7/20/2018	gypsum	3-5 mm	7, 14 MPa
QS04_004	7/27/2018	gypsum	5 mm	1, 2, 5, 7 MPa
QS04_005	7/3/2018	quartz	2.5 mm	1, 7 MPa
QS04_006	7/11/2018	gypsum	2 mm	1, 10 MPa
QS04_007	7/19/2018	talc	3 mm	1 MPa
QS04_008	7/25/2018	talc	10 mm	1, 7 MPa
QS04_009	8/24/2018	quartz	2.5 mm	1, 12 MPa
QS04_010	11/9/2018	quartz	2.5 mm	10 MPa
QS04_011	7/11/2019	30/70% talc/quartz (homogeneous mixture by weight)	5 mm	10 MPa
QS04_012	7/18/2019	talc/quartz (0 < x < 380 mm), quartz (380 < x < 760 mm)	5 mm	10 MPa
QS04_013	7/24/2019	quartz (0 < x < 570 mm), talc/quartz (576 < x < 760 mm)	5 mm	10 MPa
QS04_014	7/31/2019	talc/quartz (0 < x < 190 mm), quartz (190 < x < 760 mm)	5 mm	10 MPa
QS04_015	8/8/2019	gypsum (0 < x < 330 mm), talc/quartz (330 < x < 430 mm), gypsum (330 < x < 760 mm)	5 mm	10 MPa to compact initially, 5 MPa experiment
QS04_016	8/15/2019	gypsum (0 < x < 230 mm), talc/quartz (230 < x < 570 mm), gypsum (570 < x < 760 mm)	5 mm	10 MPa to compact initially, 5 MPa experiment
QS04_017	8/20/2019	gypsum	5 mm	10 MPa to compact initially, 5 MPa experiment
QS04_018	10/2/2019	quartz (0 < x < 230 mm), talc/quartz (230 < x < 570 mm), quartz (570 < x < 760 mm)	5 mm	10 MPa to compact initially, 5 MPa experiment
QS04_019	10/9/2019	gypsum (0 < x < 230 mm), talc/quartz (230 < x < 570 mm), gypsum (570 < x < 760 mm)	5 mm	10 MPa to compact initially, 5 MPa experiment
QS04_020	1/22/2020	quartz	5 mm	10 MPa for 1:10 mm slip, 7 MPa for 15:20 mm
QS04_021	2/14/2020	quartz	5 mm	10 MPa
QS04_022	7/2/2021	quartz	5 mm	10 MPa
QS04_023	7/6/2021	quartz	5 mm	10 MPa
